# An Updated Review on Properties and Indications of Calcium Silicate-Based Cements in Endodontic Therapy

**DOI:** 10.1155/2022/6858088

**Published:** 2022-10-30

**Authors:** Fateme Eskandari, Alireza Razavian, Rozhina Hamidi, Khadije Yousefi, Susan Borzou

**Affiliations:** ^1^Department of Endodontics, School of Dentistry, Shiraz University of Medical Sciences, Shiraz, Iran; ^2^Department of Dental Materials and Biomaterials Research Center, Shiraz Dental School, Shiraz University of Medical Sciences, Shiraz, Iran; ^3^University of Pennsylvania, College of Dentistry, Philadelphia, PA, USA

## Abstract

Regarding the common use of calcium silicate cements (CSCs) in root canal therapy, their position in the context of past and present dentistry agents can provide a better understanding of these materials for their further improvement. In this context, the present review article addresses a wide range of recent investigations in the field of CSC-based products and describes details of their composition, properties, and clinical applications. The need for maintaining or reconstructing tooth structure has increased in contemporary endodontic treatment approaches. This research thus discusses the attempts to create comprehensive data collection regarding calcium ion release, bond strength, alkalinizing activity and bioactivity, and the ability to stimulate the formation of hydroxyapatite as a bioactive feature of CSCs. Sealing ability is also highlighted as a predictor for apical and coronal microleakage which is crucial for the long-term prognosis of root canal treatment integrity. Other claimed properties such as radiopacity, porosity, and solubility are also investigated. Extended setting time is also mentioned as a well-known drawback of CSCs. Then, clinical applications of CSCs in vital pulp therapies such as pulpotomy, apexification, and direct pulp capping are reviewed. CSCs have shown their benefits in root perforation treatments and also as root canal sealers and end-filling materials. Nowadays, conventional endodontic treatments are replaced by regenerative therapies to save more dynamic and reliable hard and soft tissues. CSCs play a crucial role in this modern approach. This review article is an attempt to summarize the latest studies on the clinical properties of CSCs to shed light on the future generation of treatments.

## 1. Introduction

Calcium silicate-based cements (CSCs) are self-setting hydraulic cements encompassing mineral trioxide aggregate (MTA). CSCs are commonly used in endodontic procedures involving pulpal regeneration and hard tissue repair, such as pulp capping, pulpotomy, apexogenesis, apexification, perforation, repair, and root-end filling [1, 2]. The suitability of biomaterials used in the endodontic procedure depends on their mechanical properties, shelf life, sustainability, and above all, biocompatibility. The reported literature confirmed that biomaterials utilized in endodontics fulfilled all the mentioned requirements except for biocompatibility [3, 4]. CSC sealing ability and biocompatibility, as well as their physicochemical interaction with the local environment, are considered key determinants of their applicability in the aforementioned clinical scenarios [5, 6]. Biodentine (Septodont, Saint-Maur-des-Fosses, France), BioAggregate (Innovative Bioceramics, Vancouver, Canada) [7], EndoSequence Root Repair Material (Brasseler USA, Savannah, GA, USA), Calcium-enriched mixture cement (BioniqueDent, Tehran, Iran), Nano Fast Cement (Vista, Shiraz, Iran) [8], and TheraCal (Bisco, Schamburg, IL, USA) are examples of new commercially available CSCs. The properties and applications of calcium silicates are reviewed in this study due to their importance.

## 2. Calcium Silicate-Based Cements (CSCs)

Calcium silicate (CaSi) cements are one of the most widely used dental materials, which are also known as mineral trioxide aggregate (MTA) cements. The specifications of dicalcium and tricalcium silicates confer particular properties, leading to their extensive applications [9–13].

The first calcium silicate cement, called Portland cement, was introduced in Roman times when lime was ground with a volcanic substance in Puteoli (so-called Pozzolana). Thanks to its Pozzolana content, Roman concrete can be formed quickly even upon immersion in water, making it possible to build various buildings. After forgetting the techniques of making cement during the Middle Ages, the correct cement ratio was achieved for the second time in the 18^th^ century with the combination of clay and limestone by a British engineer named John Smithton. In 1824, Joseph Aspadin, an English mason, patented a process to produce what he referred to as Portland cement. One of the largest early buildings made of Portland-Pozzolanic cement is the first United States Great Bridge constructed in the late 19^th^ century. Currently, Portland cement is made by mixing lime, silica, alumina, and iron oxide, followed by heating. Pozzolana has been continuously used in many Portland cements [1, 14]. In the last two decades, calcium and silicate-based cements have found significant applications in modern dentistry. Mineral trioxide aggregate (e.g., ProRoot MTA, Dentsply Sirona, York, USA) and Biodentine (Septodont, St. Maur-des-Fossés, France) are some examples of silicate-based cements whose structure is based on Portland cement (calcium, iron, and aluminum silicates) [1].

To improve the clinical properties of CaSi-based cementitious materials, some additives such as modifiers, radiopaque agents, and drugs are also added (Table 1) [15–18]. Regarding the improvement of biological, mechanical, and physicochemical properties of materials at the nanoscale, their incorporation into endodontic materials like CSC could be useful [19–21].

Endodontists originally use CaSi cements as root-end filling materials. Currently, these materials are widely used in processes such as hard tissue repair and pulpal regeneration, including apexogenesis, pulpotomy, pulp capping, repair of root perforations, and root canal filling [13, 22–24] (Table 2).

The diverse capabilities of CaSi cements have led to their acceptance by researchers for their biological properties and by dentists for their excellent sealing and biocompatibility [22–24]. Novel calcium silicate-based sealants (CSBSs) are currently developed and marketed. Different products have been manufactured by companies to make the highest impact and eliminate flaws such as tough handling, long formation time, and high cost. A variety of CaSi products have been introduced based on the original formulation and/or with minor changes relative to current clinical practice [1].

The hydraulic properties of calcium silicate cements lead to their spontaneous setting upon exposure to water [25]. Moreover, they are the only dental materials that can release calcium continuously for a long time after setting in the desired location and the vicinity of water [23, 25–28]. Tricalcium silicate and dicalcium silicate are the main components of CaSi that have provided promising evidence for its widespread use in various aspects of endodontic surgery. Hydration of tricalcium silicate strongly affects the setting and establishment of initial strength, whereas dicalcium silicate hydrates much slower and offers secondary strength [29–32]. As mentioned, calcium silicate cements undergo hydration reactions upon exposure to water. The CaSi cement class (CSBS) consists of a group of premixed CSBSs requiring an external water supply and a group of two separate CSBS components with an internal water source. The reactions of both substances are identical. The first reaction is hydration observed in two different types (A, B) [33]:

  2[3CaO·SiO_2_] + 6H_2_O ⟶ 3CaO·2SiO_2_·3H_2_O + 3Ca(OH)_2_(A)
  2[2CaO·SiO_2_] + 4H_2_O ⟶ 3CaO·2SiO_2_·3H_2_O + Ca(OH)_2_(B)

This hydration reaction is followed by a calcium phosphate precipitation reaction:

7Ca(OH)_2_ + 3Ca(H_2_PO_4_)_2_ ⟶ Ca_10_(PO_4_)_6_(OH)_2_ + 12H_2_O.

The spontaneous setting of the CaSi cements mentioned earlier is the consequence of gradual hydration reaction of orthosilicate ions (SiO44–). An amorphous nanoporous calcium silicate gel forms on cement following the reaction between water molecules and calcium silicate particles. At this point, calcium hydroxide enters the pores of the gel. Over time, the CSH gel is polymerized, resulting in a strong network that improves mechanical strength [34]. Ca (OH)_2_ is released from the cement surface, making the environment more alkaline [27, 35]. The CSBS setting time under wet conditions is approximately 40–120 min. Therefore, the initial and secondary settings take 40–50 and 120–170 min, respectively [33, 36–39].

Such a long setting time is one of the main drawbacks of CSBS. The optimal setting time for many clinical applications is between 3 and 10 min. For example, apical surgery requires the shortest setting time possible due to the risk of wash-out with blood flow. Newer products such as Biodentine, MTA Plus, and light-curable TheraCal offer shorter setting times [9, 35, 36].

Thanks to their setting ability and the remarkable biological properties in wet environments (water, blood, and saliva), extensive efforts have been devoted to developing CSCs for clinical applications where other materials failed [1].

## 3. Properties of Calcium Silicates

### 3.1. Sealing Ability

The marginal sealing capability of the applied retro filling material can be exploited to inhibit bacterial growth in surgical endodontic therapy. Following apical surgery, infection and inflammation can reduce the pH level and alter the status of surrounding tissue [40]. Water-immersed CSCs can decline the setting time while enhancing the expansion and improving the sealing ability against the oily environment or even phosphate-buffered saline and fetal bovine serum. Serum proteins might be absorbed into cement, thus, decreasing the size of surface porosities [41]. Nanjappa et al. compared MTA, Biodentine, and Chitra-calcium phosphate cement (CPC) under a confocal laser scanning microscope using Rhodamine B dye with regard to sealing ability as a root-end filling material. Microleakage investigations showed a maximum of 0.45, 0.85, and 1.05 mm for Biodentine, MTA, and Chitra-CPC, respectively, suggesting the superior sealing ability of Biodentine in restoration of root-end cavities [42].

In comparison to ProRoot, MTA Biodentine and Endocem MTA considerably declined the ratio of the filled volume in acidic conditions than in the saline media. Moreover, the packing ability of Endocem MTA and Biodentine was more favorable during periapical surgery, while high adhesion of ProRoot MTA to the instrument resulted in its coming out, even after being packed into the cavity [43].

**Figure 1:**
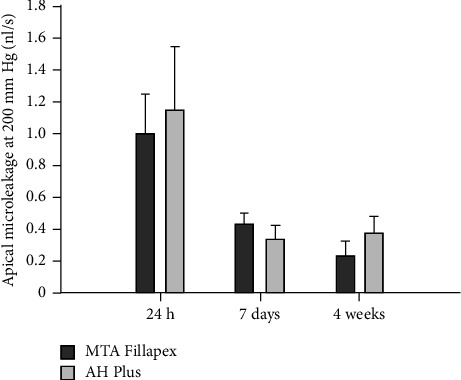
A bar chart of the mean apical microleakage measurements for both trial groups after 1 day, 1 week, and 4 weeks. The error bars demonstrate standard deviation from the mean value [46].

Numerous methods and devices have been developed to assess apical or coronal microleakage, namely, fluid filtration, dye extraction and dye penetration, bacterial leakage models, and protein leakage model. Among the mentioned methods, the fluid filtration system has shown great promise in endodontics [44]. A fluid filtration approach enables quantitative measurement of root canal leakage. Moreover, leakage could be frequently measured in a specific duration in the absence of any root specimen destructions. Evidence proved that a calcium silicate-based sealer (MTA Fillapex) had higher sealing capability than an epoxy resin sealer (AH Plus) by utilizing a fluid infiltration approach. Some characteristics of MTA Fillapex such as a water absorption property could be advantageous in providing appropriate sealing because it facilitates slight expansion in the material (Figure 1) [46].

**Figure 2:**
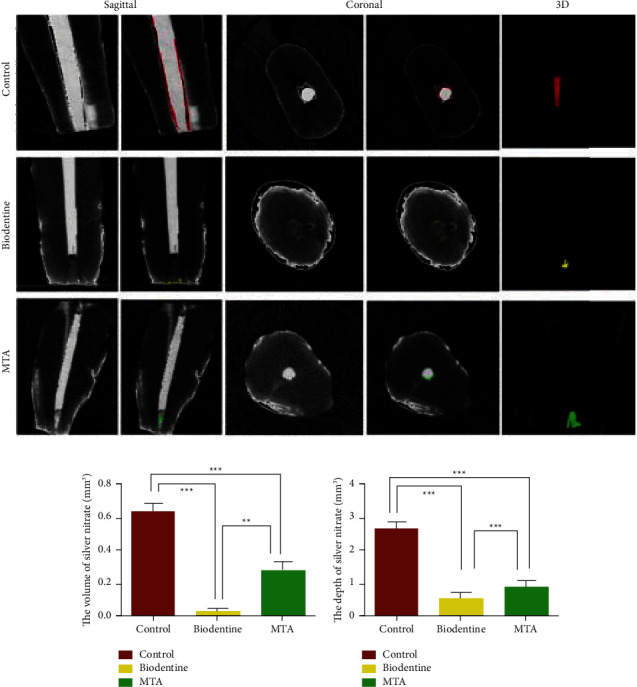
Microleakage evaluation by a micro-CT analysis. (a) Micro-CT images representing the silver nitrate solution volume which infiltrated root canals in each of the studied groups. (b) Quantitative assessment of nitrate solution volume which infiltrated root canals. (c) Quantitative assessment of nitrate solution depth which infiltrated root canals. ^*∗∗*^*P* < 0.01 and  ^*∗∗∗*^*P* < 0.001 [48].

Dye penetration is another simple technique that can evaluate microleakage [46]. In addition to being inexpensive and nontoxic, dye penetration is the most widely used method, and it is also able to detect low concentrations of pollutants [47]. Recently, it has been shown that Biodentine attenuated penetration of dye while providing perforation repair through furcation and enhancing its sealing capability against the resin-modified glass ionomer cement group and ProRoot MTA. Despite a statically insignificant difference between the ProRoot MTA group and the Biodentine group [46], an investigation estimated the sealing capability of Biodentine and ProRoot MTA by infiltrated nitrate solution with a microcomputed tomography analysis which reported better outcomes for Biodentine. This procedure offers extraordinary benefits such as complete three-dimensional fidelity that enables the quantitative interfacial adaptation assessment in any direction and location (Figure 2) [48].

### 3.2. Compressive Strength

As one of the prime physical characteristics of hydraulic cements, compressive strength mainly indicates the hydration method and settings of hydraulic cements. To be applied in vital pulp treatments, cement must be able to endure indirect masticatory forces and impede the set cement failure. Moreover, no changes must occur in physical properties due to acid etching before the placement of composite restoration [48]. The acid etching process [49], as well as saliva and blood from the oral cavity [50], had no negative impacts on Biodentine and ProRoot MTA compressive strength, and these calcium silicate-based cements seem to be better options.

Moreover, no negative impact was observed in the compressive strength of MTA and Biodentine upon their exposure to saliva and oral cavity blood [51].

Some studies stated considerably higher compressive strength of ProRoot MTA and Biodentin than that of MTA Angelus [52, 53]; this physical property can be enhanced with mechanical mixing through an amalgamator and ultrasonic agitation (Table 3) [50].

### 3.3. Push Out Bond Strength

Noteworthy, an endodontic biomaterial should properly adhere to root canal dentine to provide sufficient assistance for preserving the integrity of the root filling-dentine interface against inert conditions and resisting the filling material luxation in the course of operative practice. Concerning bond strength, this attribute can be assessed in vitro through the push-out test. Prior addition of calcium hydroxide to distilled water intracanal placement can raise dispossession calcium silicate cements resistance [54], although smear layer elimination is harmful to bond strength [55]. A Contemporary calcium silicate-based sealer establishes rapid and easier obturation and displays appropriate bond strengths [55]. Additionally, the use of various chelating agents did not impress calcium silicate-sealer push-out bond potency [56].

Quick-setting MTA and pozzolan-based cements (ENDOCEM MTA and ENDOCEM Zr) have been recently developed with less tooth discoloration in comparison with MTA [57]. Besides, they showed an appropriate bond strength performance comparable to the bond strength of white MTA [58].

### 3.4. Radiopacity

Cement enhancement and its distinguishability from enclosing anatomical structures require the incorporation of radio-pacifying material into calcium silicate-based cements. This makes cement identifiable during radiographic procedures [59]. As an opacifier, bismuth oxide is mixed with white and gray ProRoot MTA and MTA Angelus [60]. Meanwhile, numerous investigations have demonstrated that bismuth oxide could have negative impacts on biocompatibility [61] and physical characteristics of the system [62]. Recent calcium silicate-based cement, named Biodentine, includes zirconium oxide as a radiopacifier although its radiopacity was lower than ProRoot MTA [63, 64]. The EndoSequence BC sealer encompasses the same opacifier with lower radiopacity than the AH Plus sealer [65].

### 3.5. Setting Time

Calcium-silicate cements suffer from the long setting time, particularly when utilized as a root-end filling material to provide structural durability of the reconstruction and supply appropriate potency to elude luxation during restorative processes [66]. Diverse additives have been employed to speed up the setting time of MTA [67, 68]. The incorporation of 1% methylcellulose and 2% calcium chloride into ProRoot MTA leads to ∼30% faster setting time [69]. Moreover, an amalgamation of 8% and 10% of nano-SiO_2_ to MTA accelerated the hydration process and reduced the setting time with no adverse impacts on the compressive and flexural strength of MTA [70]. Some recently commercialized types of calcium silicate cements (e.g. Biodentine and Endocem MTA) displayed shorter setting time than ProRoot MTA which can result in clinical advantages in vital pulp therapy procedures, declining provisional restoration leakage and eliminating the need for the 2^nd^ appointment for complete restoration [41, 71]. Calcium chloride available in Biodentine liquid serves as a setting time accelerator and water-reducing agent [72].

### 3.6. Calcium and Ion Release

Ionic dissolution-manufactured particles have been regarded as crucial elements of the biological activities of calcium silicate-based materials [73]. The hard tissue formation action of MTA can be assigned to its ability to release Ca^2+^ and create alkaline pH [36]. Higher contents of calcium and silicon ions may provide osteoblasts to develop and differentiate and also produce high pH environments, further promoting the periapical healing processes [74]. In apical surgery and perforation repair, calcium silicate cements may confront an acidic environment; therefore, some studies measured their ion release at low pH and showed that bioactive materials released higher amounts of Ca^2+^ at lower pH [68, 75].

### 3.7. Alkalinizing Activity

Due to the dehydration procedure, MTA cements are known to possess high alkaline pH. Hydration reactions are mainly due to entailing dicalcium and tricalcium silicate cements during setting time [76]. Their antibacterial or bacteriostatic activity can also be attributed to the alkaline pH, which makes an undesirable situation for any remaining bacteria [77]. The pH of ProRoot MTA was 10.5 and 12.9 after 3 hours, which was similar to calcium hydroxide with adequate antimicrobial activity [23]. Compared to ProRoot MTA, the pH of MTA Angelus was moderately higher [78]. Moreover, MTA-based sealers showed higher values of pH than resin-based sealers [77]. The alkaline property of Bioceramic sealers for an extended period of time enhanced the solubility. This property of the Bioceramic sealer can promote its biological and antibacterial outcomes, whereas its constant solubility may affect its ability to impede apical leakage [79]. The minimum pH of MTA Fillapex sealers was nearly 7.5 after 3 and 24 hours, which rose over time [80]. Both iRoot SP and EndoSequence BC Sealers showed high pH values [81]. Furthermore, Endoseal exhibited higher alkalinity than AH Plus [28].

### 3.8. Porosity

**Figure 3:**
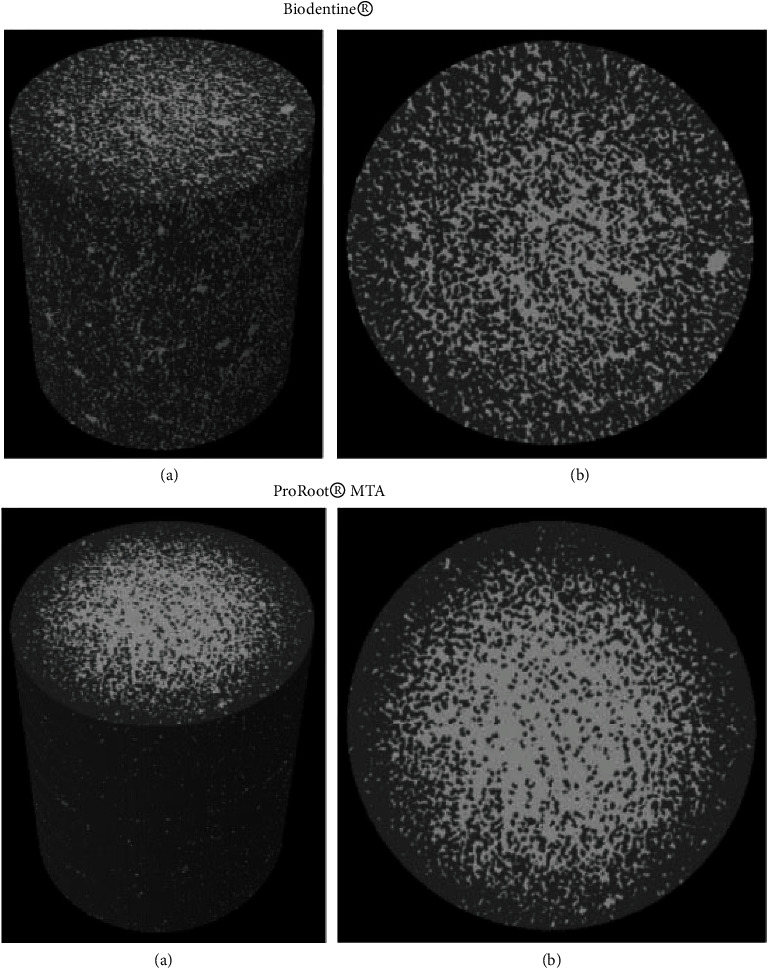
Micro-CT graphs indicating porosity of Biodentine® and ProRoot® MTA. (a) A 3D model in the presence of endodontic material and porosity. (b) A cross section demonstrating repair material and porosity of Biodentine® and ProRoot® MTA. Pores are marked with the white color, while repair materials without porosity appear in gray.

The porosity may be beneficial to the hydration procedure of HCSCs and their capacity to release bioactive ions [82]. There is a moderate negative association between the porosity and flexural strength and sealing ability, which weakens materials [83–85]. The porosity of the mixture increased by enhancing water proportion [69]. Comparing manual mixing with mechanical mixing in terms of total porosity and flexural strength, mechanical mixing of encapsulated cements showed considerable advantages over manual mixing [86]. Biodentine attains less porosity than ProRoot MTA, as demonstrated by micro-CT (Figure 3) [87]. No significant differences were observed between the other new calcium silicate cements and MTA [88, 89].

**Figure 4:**
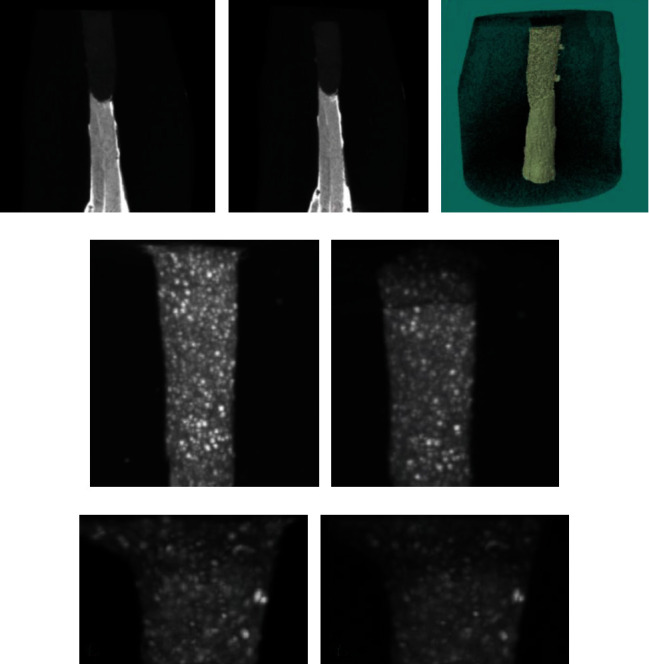
The volume change of Biodentine in acidic pH. Before scanning with micro-CT, Biodentine was fully set on the retrograde cavity (a). The volume loss after exposure to acidic pH (b, c). Density variation of ProRoot MTA observed by the CTVox programme. Before and after exposure to the acidic medium (d, e). A loss of density was observed. After exposure to acidic pH, internal changes in MTA Angelus were observed by the CTVox programme. In acidic pH, larger particles converted into smaller ones, which increased the gaps (f, g).

Unlike other media, acidic pH significantly reduced the volume of ProRoot MTA, MTA Angelus, and Biodentine. Biodentine exhibited the highest loss of volume and density after treatment in an acidic medium. Porosity formation in the acidic medium was observed in Biodentine amongst all materials. The three-dimensional structures of all materials changed upon exposure to acidic pH, while fewer changes occur in the structures of materials treated with blood and alkaline materials (Figure 4) [90].

### 3.9. Solubility

As dissolution of materials could result in their leakage and thus, failure of the treatment, CSCs should have low solubility in body fluids [88, 91]. Several researchers reported a rise in solubility and porosity upon increasing the amount of water in the mixture [92, 93]. According to ISO 6876:2001, solubility of Biodentine and ProRoot MTA is below 3%, i.e., an acceptable range of weight loss for the solubility test. However, in extended duration, solubility of Biodentine considerably surpassed that of ProRoot MTA [85, 94–96]. An investigation carried out by Gandolfi et al. led to some conflicting outcomes as the solubility of Biodentine considerably falls behind the solubility of ProRoot MTA, MTA Angelus, and MTA Plus [95]. However, TheraCal showed very low solubility; the lowest solubility belongs to ProtRoot MTA due to its high resin content [91]. On the other hand, because of inadequate biocompatibility and marginal adaptation insufficiency, TheraCal fails in changing MTA in furcation perforation repair [68]. Silva et al. performed a systematic review and meta-analysis in 2021 and assessed whether epoxy resin-based root canal sealers have higher solubility than calcium silicate-based root canal sealers. The meta-analysis demonstrated the lower solubility of AH Plus. Compared to Bio-C sealer, BioRoot RCS, MTA Fillapex, Plus, and TotalFill BC sealer, AH Plus displayed lower solubility [97].

### 3.10. Bioactivity

Calcium silicate-based cements can stimulate the formation of hydroxyapatite as a bioactive property [98]. Apatite formation has been investigated by in vitro and in vivo studies [10, 99, 100]. Moreover, portlandite dissolution (calcium hydroxide) and calcite production (which is calcium carbonate, generally the product of Ca(OH)_2_ interaction with atmospheric CO_2_) are noticed on setting white Portland cement interaction with simulated body fluids [101]. The apatite layer could be an optimum environment to substantiate new bone establishment via differentiation and colonization of stem cells and osteoblasts. The major clinical results of MTA cements are demonstrated by the synchronization of apatite and epigenetic signals to release ions. Moreover, the presence of apatite on the cement surface is related to cell growth and cell differentiation [102–104].

The evaluation of ALP activity (alkaline phosphatase activity) determines the induction of mineralized tissue formation in calcium silicate-based sealers (TotalFill BC sealer) and cements (Biodentine and MTA Plus) during exposure to human osteoblast-like cells [105, 106]. CeraSeal and EndoSequence BC sealers show gene expression and mineralization ability [107].

**Figure 5:**
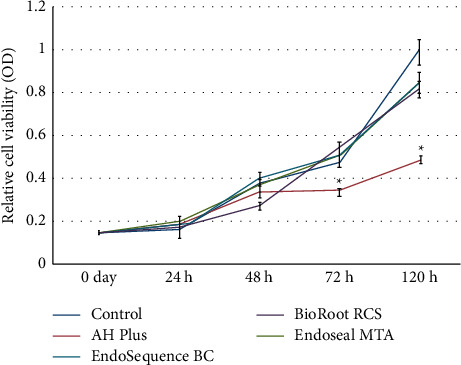
Relative cell viability rate based on the methylthiazolyldiphenyl-tetrazolium (MTT) assay. Statistically remarkable differences among the control and trial groups are shown by asterisks.

**Figure 6:**
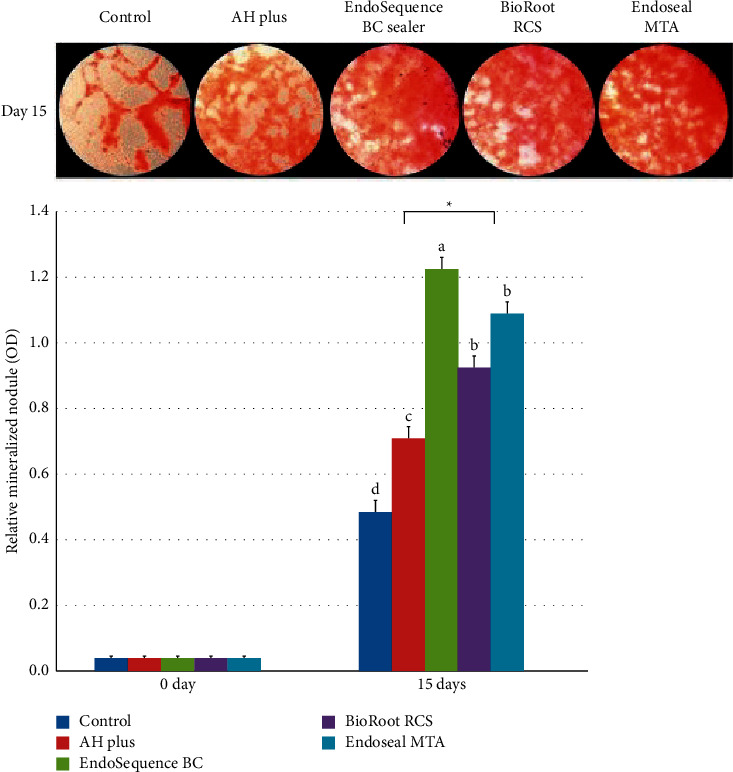
Relative mineralized nodule formation rate based on the alizarin red staining assay. Statistically significant differences between the control and trial groups are shown by asterisks. Dissimilar superscript letters denote statistically significant differences.

Numerous in vitro and in vivo studies indicated the biocompatibility and lower cytotoxicity of MTA and new CSCs; none of these materials triggered a severe inflammatory response [102, 105, 108–110]. Better biological and mineralization characteristics of calcium silicate-based root canal sealers were confirmed in an in vitro study while comparing them with conventional resin-based sealers (Figures 5 and 6) [111]. Besides, a recent systematic review and meta-analysis stated that concerning biocompatibility, bioactivity, and genetic expression regarding SHEDs, MTA, Biodentine, EndoCem Zr, RetroMTA, and iRoot BP are all suitable for the treatment of vital pulp in the primary teeth, indicating their clinical applicability [112].

### 3.11. Tooth Discoloration

The tooth discoloration mechanism is mostly theorized based on oxidation due to the presence of heavy metal oxides (i.e., iron or bismuth) in cements [113]. Studies have also indicated that blood-contaminated CSCs lead to color change due to the penetration of erythrocytes into the tooth structure and entrapment of blood components. More recent agents containing zirconium oxide as a contrasting substance did not show great staining potential [114, 115]. Besides, studies showed that the replacement of bismuth oxide with zirconium oxide (as a radiopacifier alternative) can decrease discoloration. In the absence of blood, ENDOCEM Zr and RetroMTA (which contain zirconium oxide) demonstrated lower tooth discoloration than ProRoot MTA and MTA Angelus (which encompass bismuth oxide) [116–118]. In the presence of blood, however, no considerable difference was observed among ProRoot MTA, Biodentine, OrthoMTA, and ERRM [119]. The radiopacifying material of BioAggregate is tantalum peroxide rather than bismuth oxide, which reduces tooth discoloration compared to ProRoot MTA. However, it caused higher levels of tooth discoloration compared to Biodentine. This trend can be attributed to higher porosity and greater fluid uptake of BioAggregate [120]. A systematic review by Możyńska et al. reported that Ortho MTA (BioMTA, Seoul, Korea), gray and white ProRoot MTA (Dentsply, Tulsa, OK), and gray and white MTA Angelus (Londrina, PR, Brazil) demonstrated the staining potential. Although MTA Ledermix (Riemser Pharma GmbH, Greiswald-Insel Riems, Germany), MM-MTA (Micro Mega, Besancon Cedex, France), Odontocem (Australian Dental Manufacturing, Brisbane, Australia), EndoSequence Root Repair Material (Brasseler USA, Savannah, GA), Portland cement, Retro MTA (BioMTA), and Biodentine (Septodont, Saint-Maur-des-Fosses, France) showed the smallest staining potential through individual studies [61].

## 4. Clinical Applications of Calcium Silicate Cements

### 4.1. Root Perforation Repair Materials

Iatrogenic or pathologic communication among the root canal system and the external root surface is described as perforation [100]. Root perforations represent an unfavorable predicament of root canal therapy and necessitate urgent treatment in order to hinder contamination of periodontal tissue and resorption of alveolar bone. Nature of the perforation repair material is a prognostic factor relating to this procedural complication [121]. Various materials have been proposed for repairing perforations; notwithstanding, none of them meet all ideal criteria, and the choice of the repair material is still a challenge [100, 121]. It was revealed that two calcium silicate-based cements, MTA and Biodentine, result in appropriate periradicular tissue responses as root perforation materials, and in 30% of the samples, cementum repair had occurred [122]. Another report stated that tissue response following implementation of MTA and Biodentine was agreeable, and both of them formed mineralized tissue resulting in partial reinsertion of fibers. Interestingly, application of MTA could bring about expression of proteins, which are related to the formation of a cementum-like mineralized tissue formation [123]. A micro-CT evaluation of furcation perforation repair elucidated that radiographic response of MTA and Biodentine was equal, and they showed equivalent resorption of hard tissue and repair. Although, in comparison to MTA, Biodentine resulted in remarkably less inflammation and superior cement repair, its volume of extruded material was less [124]. In addition, the sealing ability of the repairing material is a critical factor for the purpose of blockage of continuing contamination. The sealing ability and handling properties of Biodentine and EndoSequence were superior to MTA Angelus for repairing perforation of furcations [125]. Interestingly, Solanki et al. summed up their systematic review study that its favorable biological properties along with its good sealing ability make Biodentine a competent retrograde filling material for clinical use [126]. Besides, CEM cement demonstrated more favorable sealing of furcation perforations compared to MTA [127].

In order to overcome the flaws of available materials, many investigations are performed to introduce more promising materials [100]. On that account, one of the novel materials for repairing of furcation perforations, NeoMTA Plus, has been examined recently regarding its biocompatibility. It was reported that early biocompatibility of NeoMTA Plus was superior to MTA Angelus following 1 week of delayed repairing of furcation perforation; however, the late biocompatibility after 1 and 3 months was similar [121].

### 4.2. Pulpotomy

Contemporary, minimally invasive methods like partial and full pulpotomy have obtained great acceptance for management of carious exposure of teeth to preserve the vitality of the dental pulp [128]. A recent movement towards application of MTA in vital pulp therapies has been observed, and many investigations have been performed [129]. A 1-year follow-up study reported that treatment outcomes of partial pulpotomy with ProRoot MTA, OrthoMTA, and RetroMTA was satisfactory, and no significant difference was observed between these materials regarding clinical and radiographic evaluations [129]. Interestingly, MTA pulpotomy of cases showing clinical signs of irreversible pulpitis and the presence of periapical radiolucency resulted in acceptable treatment outcomes with success rates of 84% and 76%, respectively [130]. A recent investigation has assessed the efficacy of MTA and Biodentine as pulpotomy agents in carious-exposed vital immature mandibular first permanent molars, and both of them demonstrated a high success rate [131]. Besides, partial pulpotomy by ProRoot MTA and Biodentine demonstrated successful outcomes in permanent teeth with irreversible pulpitis in patients of age ranging between 6 and 18 years However, gray discoloration following Biodentine usage was lower in comparison to ProRoot MTA [132]. Also, MTA and Biodentine were considered as two suitable pulpotomy materials for primary teeth, which were exposed due to a carious lesion, and their long-term retention was crucial [133]. In addition, long-term follow ups revealed desirable biocompatibility and antibacterial activity of ProRoot MTA, OrthoMTA, and RetroMTA in partial pulpotomy of permanent teeth with no remarkable statistically difference among them [134]. A histological comparison between ProRoot MTA and RetroMTA as partial pulpotomy agents manifested pulp disorganization, absence of inflammatory response, and discontinuous mineralization following application of RetroMTA, and in spite of the shorter setting time of Retro MTA, this pulpotomy material was less favorable than ProRoot MTA [135]. Another investigation has compared Biodentine and MTA as pulpotomy materials in the treatment of traumatized immature anterior permanent teeth. Clinical and radiographic outcomes of both materials were equivalent; however, prevalence of discoloration was higher following usage of MTA [136]. Regarding postoperative pain consequent to pulp therapy, pulpotomy with MTA, CEM, and RCT revealed satisfying relief of postoperative pain [137, 138]. A recent research study, which has compared the efficacy of pulpotomy with diode lasers and MTA and MTA alone, stated that incorporation of diode lasers in the pulpotomy procedure promoted the success rate of the treatment and has offered further investigations on the capability of lasers for advancement of pulpotomy treatments [139].

### 4.3. Apexification

One of the materials of the choice for apexification is MTA due to its various desirable properties [140]. Apexification with MTA had demonstrated favorable clinical and radiographic outcomes in the open apex teeth [141]. It was reported that apexification and revascularization by MTA can retain the function of the teeth and also result in the resolution of the disease [142]. In addition, regardless of the prior calcium hydroxide usage, applying MTA as apical plug-favored apexification and healing of periapical pathosis [143]. A comparative study reported that following apexification with MTA, barrier was formed in 90.90% of cases, while application of calcium hydroxide led to barrier formation in 81.81% of cases. Moreover, in cases of immediate obturation of immature roots with wide-open apices, application of MTA seemed promising [144]. Another report stated that in comparison to NeoMTA Plus, biomineralisation capability of ProRoot MTA and Biodentine was superior, and during the first month after application of these two materials, a positive influence on the fracture resistance of dentine was observed [145].

### 4.4. Direct Pulp Capping

Direct pulp capping (DPC) is performed when iatrogenic or traumatic injury has exposed a healthy pulp [146]. MTA, calcium hydroxide, and Biodentine are the frequently used materials for DPC [147]. Paula et al. conducted a systematic review and meta-analysis regarding the effectiveness of biomaterials and techniques used for direct pulp capping. Their results indicated that compared to calcium hydroxide cements, a higher success rate was shown for MTA cements in all parameters. Also, a lower inflammatory response and more predictable hard dentin barrier formation were found in MTA cements compared to calcium hydroxide cements [148]. A recent study has assessed formation of the reparative dentine bridge following application of Biodentine as a direct pulp capping agent. It was concluded that Biodentine is capable of induction of reparative dentin formation in direct pulp capping, which was assessed through CBCT imaging modality [149]. It is worth mentioning that the direct pulp capping material influences the volume of reparative dentin bridge formation. A report stated that the reparative dentin bridge formation after application of MTA and Biodentine was remarkably superior in comparison to single bond universal [150]. Also, another report recommended usage of MTA and Biodentine as substitutes of calcium hydroxide in direct pulp capping [151]. A comparative study on ProRoot MTA and Biodentine demonstrated that Biodentine established a noninferior successful outcome as a direct pulp capping agent for cariously exposed permanent teeth of 6 to 18-year-old patients. Furthermore, the possible use of Biodentine was suggested in the esthetic zone since it did not lead to grayish tooth discoloration [152].

Lately, the efficacy of novel calcium silicate cement, nominated as Protooth, in direct pulp capping of primary molars has been evaluated. The results indicated that this novel cement has favorable properties as a direct pulp capping material, such as biocompatibility, hydrophilicity, fast setting time, and adequate tensile strength, which make this agent promising for implementation in pediatrics [153].

### 4.5. Root-End Filling Material

Recently, an investigation evaluated marginal adaptation of MTA, Biodentine, and amalgam. SEM analysis indicated favorable marginal adaptation of all of these root-end filling agents; however, MTA and Biodentine were superior to amalgam [154]. Another recent study has assessed 4 different commercial root-end filling materials and concluded that in comparison to ProRoot MTA, physicochemical and mechanical characteristics and cytocompatibility of Endocem MTA, RetroMTA, and DiaRoot Bio MTA are satisfactory, and these 3 root-end filling materials are recommended as auspicious alternatives to commonly used MTA [155]. *μ*CT evaluation of physicochemical behavior of 3 retrograde filling materials over time exhibited dimensional and volumetric stability of MTA and IRM. Baseline values of porosity and adaptability of Biodentine were satisfactory, but following immersion in PBS, its thickness was reduced, and its porosity and interface voids were increased [156]. Another *μ*CT assessment compared MTA, zinc oxide-eugenol cement, and Biodentine regarding their physicochemical characteristics. Dimensional change in zinc oxide-eugenol cement was higher among the experimental groups, and the solubility of Biodentine was greater after 1 week. The filling ability of Biodentine and zinc oxide-eugenol was higher than MTA. Besides, after 1 month, volumetric changes in Biodentine were more than in MTA [157]. In another experiment, a confocal laser scanning microscope was used to make a comparison between silver amalgam, resin-modified glass ionomer cement, Cermet cement, Biodentine, and MTA Angelus, regarding their sealing ability as root-end filling materials. It was reported that the most superior sealing ability was seen in the Biodentine group, followed by MTA, Cermet cement, resin-modified glass ionomer cement, and silver amalgam, respectively [158]. Currently, the utilization of nanotechnology in dentistry is an emerging field where incorporation of nanoparticles in various filling materials have enhanced their physicochemical characteristics, strength, shelf life, longevity, and biocompatibility. In addition, these filling materials are prepared via biogenic routes thus, showing their cost-effective nature as well [4, 159, 160].

A hermetic seal was created to reduce microleakage influences on the outcome of endodontic therapy. Microleakage of Biodentine and BioAggregate was reported to be lower than that of MTA Plus and MTA, and as a result, Biodentine and BioAggregate were advised as more favorable retrograde filling materials [161]. Another comparison mentioned that microleakage of Biodentine and MTA were lower than that of glass ionomer cement as retrograde cavity filling materials [162].

### 4.6. Root Canal Sealers

A research study on cell viability and cell migration capability of EndoSequence BC sealer, BioRoot RCS, and Endoseal MTA demonstrated favorable results of these 3 calcium silicate-based root canal sealers in comparison to the control group. Also, in comparison to AH Plus sealers, mineralization activity was promoted following the usage of calcium silicate-based sealers [117]. Regarding the retreatability of the sealers, a comparative study between EndoSeal MTA, EndoSequence BC sealer, and AH Plus displayed that the highest amount of remaining sealers belonged to EndoSeal MTA, particularly in C-shaped root canals [163]. A comparison between BioRoot RCS, MTA Fillapex, Endo CPM, and AH Plus sealer revealed that retreatability of calcium silicate-based sealers was more favorable than AH Plus sealers, and the amount of remaining sealers was lower. The time required for retreatment was shorter [164]. Furthermore, the sealer penetration depth of BioRoot RCS is higher in all root sections in comparison to AH26 sealer. Besides, the penetration depth of BioRoot RCS was greater than MTA Fillapex in the apical third in retreatment procedures [165].

Biocompatibility and bioactive capability of MTA Fillapex and NeoMTA Plus have been investigated recently. These two calcium silicate-based sealers demonstrated desired biocompatibility since the primary moderate inflammatory response was replaced by thin fibrous capsules, elucidating structural reorganization of the connective tissue around these sealers over time. The observed inflammatory response lowered more quickly in the NeoMTA Plus group than in the MTA Fillapex group. Also, two of the sealers revealed favorable bioactive capability [166]. Cytocompatibility of TotalFill BC sealers, a newly introduced bioceramic sealer, was found superior to MTA Fillapex and AH plus. However, adhesion, proliferation, migration, and viability of hPDLSC reduced following application of MTA Fillapex. Besides, MTA Fillapex demonstrated cytotoxicity, which was significantly higher than that of BC sealers [167]. Moreover, Ca2^+^ ion release and pH of TotalFill BC sealers was more than those of MTA Fillapex and AH Plus sealers. On the other hand, MTA Fillapex presented higher values of volumetric changes than TotalFill BC sealer and AH plus sealer [168]. Postoperative pain after root canal therapy is another crucial factor, which influences patients' satisfaction. A comparison between Endoseal MTA, EndoSequence BC, and AH Plus sealer demonstrated equal postoperative levels after usage of these sealers in endodontic treatment [169]. No remarkable difference was shown between MTA Fillapex, Endofill, and AH Plus sealer regarding postoperative pain following root canal obturation [170].

### 4.7. Management of Root Resorptions

Root resorptions are classified into two categories including external and internal root resorptions [171]. Generally, internal root resorption is a result of trauma or chronic inflammation, and its characterization is dentine progressive destruction along the root canal walls [172]. Undetected and untreated internal root resorption can grow larger and ultimately may perforate the root from inside, which can make its treatment more challenging. It was demonstrated that a preferable material for repairing perforating internal root resorptions may be backfilled using calcium silicate cements (Biodentine, MTA, MTA Plus), compared to a combination of gutta-percha and sealer [173]. Considering void formation following obturation of internal root resorptions, a comparison was performed between Biodentine, MTA, TotalFill BC root canal sealer (bulk-fill form), and warm gutta-percha with TotalFill BC sealer. None of these obturation materials were detected without void formation; however, Biodentine demonstrated the least void formation in comparison to the other experimental groups [174]. Another report stated that obturation of artificial internal root resorptions resulted in an optimum outcome using EndoSeal MTA with a warm vertical technique and a single-cone technique rather than a single-cone technique using GuttaFlow 2 and EndoSequence [175]. Also, release of calcium ion, high pH, and favorable root reinforcement capacity were observed in inflammatory resorption obturated with Bioceramic sealers like iRoot SP and MTA Fillapex. So, an initial medication with calcium hydroxide over a period of 7 days followed by obturation with gutta-percha and bioceramic sealer was recommended as an alternative and beneficial treatment modality in comparison to long-term application of calcium hydroxide [176]. Interestingly, another study reported that management of inflammatory root resorptions with MTA may be considered as a desirable treatment option due to the observed diffusion of calcium ions through defects in the dentine obturated with MTA [171]. Moreover, after one month's placement of MTA in simulated external inflammatory root resorptions, a higher pH value was recorded in comparison to calcium hydroxide placement [177].

## 5. Conclusion

Indications of CSCs have been widely supported by clinical studies due to their favorable properties reviewed in this paper. Higher sealing ability and lower solubility contributed to their wide range of applications in regenerative dentistry. Thorough knowledge on different aspects of CSCs will assist in their successful implementation. Accordingly, further development of CSCs can significantly modify their physical properties and expand their clinical application.

## Figures and Tables

**Table 1:** Calcium silicate-based cements used in endodontics [15–18].

Name of ingredients (%)	Portland cement	Biodentine	MTA	Angelus MTA	White MTA	Nano WMTA	BioAggregate	Grey MTA	Grey Angelus MTA
Calcium silicate oxides (Ca_3_SiO_5_ and Ca_2_SiO_4_)	94.9	80.1	75.6	74.5	34.1	65	65	30.3	30.1
Magnesium phosphate (Mg_3_(PO_4_)_2_)	—	—	—	—	0.9	—	—	2.3	—
(Bi_2_O_3_)	—	—	21.6	14.0	56.7	17	—	58.8	38.8
Calcium carbonate (CaCO_3_)	—	14.9	—	—	0.9	—	—	—	3.9
Calcium phosphate (Ca_3_(PO_4_)_2_)	—	—	—	—	1.6	—	6	1.0	*∗∗∗*
Calcium silicate (Ca_2_SiO_4_)	—	—	—	—	1.7	—	—		1.0
Calcium magnesium aluminum (Ca_2_MgO. 2AlFeO. 6SiO.2O_5_)	—	—	—	—	—	—	—	2.9	4.2
Barium zinc phosphate (BaZn_2_(PO_4_)_2_)	—	—	—	—	—	—	—	—	3.4
Tantalum pentoxide (Ta_2_O_5_)	—	—	—	—	—	—	25	—	—
Silicon oxide (SiO_2_)	—	—	—	0.5	—	—	4	—	—
Zirconium oxide (ZrO_2_)	—	5.0	—	—	—	—	—	—	—
Tricalcium aluminate (Ca_3_Al_2_O_6_)	0.8	—	—	2.0	—	4	—	—	—
Calcium oxide (CaO)	—	—	—	8.0	—	—	—	—	—
Strontium carbonate (SrCO_3_)	—	—	—	—	—	3	—	—	—
Gypsum (CaSO_4_•2H_2_O)	—	—	—	—	—	5	—	—	—

**Table 2:** Types of HCSCs and some calcium hydroxide materials for the endodontic process [13, 22–24].

Materials	Uses	Manufacturer	Composition
Biodentine	PC, Ag, REA, RP	Septodont, Saint-Maur-des-Fossés, France	Powder: tricalcium silicate
			Liquid: aqueous calcium chloride solution and excipients
Harvard MTA Caps	PC, Ag, REA, RP	Harvard Dental International GmbH, Hoppegarten, Germany	Powder: mineral trioxide aggregate and bismuth oxide

Ledermix MTA	PC, Ag, REA, RP, Af	Riemser, Riems, Germany	Powder: various mineral oxides
			Liquid: water

MM-MTA	PC, Ag, REA, RP, Af	Micromega, Besancon, France	Powder: tricalcium and dicalcium silicate, calcium carbonate
			Liquid: H_2_O

MTA Angelus	PC, Ag, REA, RP, Af	Angelus dental solutions, Londrina, PR, Brazil	Powder: SiO_2_, K_2_O, Al_2_O_3_, Na_2_O, SO_3_, CaO, Bi_2_O_3_, MgO, insoluble CaO, KSO_4_, NaSO_4_, and crystallized silica
			Liquid: H_2_O

MTA Plus	PC, Ag, REA, RP, A, RCS	Prevest DenPro Limited, Jammu, India	Powder: tricalcium and dicalcium silicate
			Liquid 1: H_2_O
			Liquid 2: gel

ProRoot MTA	PC, Ag, REA, RP, Af	Dentsply tulsa, Johnson City, TN, USA	Powder: white Portland and bismuth oxide
			Liquid: H_2_O

Tech Biosealer capping	PC, Ag	Isasan srl, Rovello porro, Co, Italy	Powder: mixture of white CEM, calcium sulfate, calcium chloride, and montmorillonite
			Liquid: DPBS (Dulbecco's phosphate buffered saline)
TheraCal	PC, Ag	Bisco Inc, Schaumburg, IL, USA	Paste: 45 wt.% mineral material (type III Portland cement), 10 wt.% radiopaque component, 5 wt.% hydrophilic thickening agent (fumed silica), and 45% methacrylic resin

BioAggregate	RP	Innovative BioCeramix Inc, Vancouver, Canada	Powder: aluminum-free calcium silicate, monocalcium phosphate, and tantalum oxide
			Liquid: deionized water

EndoSequence Bioceramic	REA, RP	Brasseler, Savannah, GA, USA	Powder: calcium silicate, calcium phosphate monobasic, calcium hydroxide, zirconium oxide, tantalum oxide, filler, and thickening agents
			Liquid: H_2_O

Retro MTA	REA, RP	BioMTA, Seoul, Republic of Korea	Powder: tricalcium silicate, dicalcium silicate, tricalcium aluminate, tetracalcium aluminoferrite, free calcium oxide, and bismuth oxide
			Liquid: deionized water

Tech Biosealer root-end	REA, RP	Isasan srl, Rovello Porro, Co, Italy	Powder: mixture of white CEM, calcium sulfate, calcium chloride, bismuth oxide, and montmorillonite
			Liquid: DPBS (Dulbecco's phosphate buffered saline)
iRoot SP, iRoot BP and iRoot BP Plus	RCS, Af	Innovative BioCeramix Inc, Vancouver, Canada	Paste: aluminum-free calcium silicate, calcium phosphate, calcium hydroxide, niobium oxide, and zirconium oxide
MTA Fillapex	RCS, Af	Angelus dental solutions, Londrina, PR, Brazil	Paste: salicylate resin, diluting resin, natural resin, bismuth trioxide, nanoparticulated silica, MTA, and pigments

Ortho MTA	RCS, Af	BioMTA, Seoul, Republic of Korea	Powder: tricalcium silicate, dicalcium silicate, tricalcium aluminate, tetracalcium aluminoferrite, free calcium oxide, and bismuth oxide
			Liquid: deionized water

Tech Biosealer Endo	RP, RCS, Af	Isasan srl, Rovello Porro, Co, Italy	Powder: mixture of white CEM, calcium sulfate, bismuth oxide, montmorillonite, and sodium fluoride
			Liquid: DPBS (Dulbecco's phosphate buffered saline)

**Table 3:** Minimum, maximum, mean, and standard deviation of the compressive strength of the groups [50].

	MTA type	Mixing/placement technique	Mean	SD	Minimum	Maximum
G1	ProRoot	MM + US	101.71	18.64	81.90	129.41
G2	ProRoot	MM	90.85	25.25	50.69	125.22
G3	ProRoot	Man M + US	90.78	33.60	36.88	147.26
G4	ProRoot	Man M	90.77	27.21	58.86	143.57
G5	Angelus	MM + US	81.36	24.94	50.97	124.03
G6	Angelus	MM	74.14	28.43	30.61	117.86
G7	Angelus	Man M + US	54.96	17.47	32.55	81.10
G8	Angelus	Man M	53.47	22.31	24.75	89.64

Man M: manual mixing; MM: mechanical mixing; US: ultrasonication.

## Data Availability

The data supporting this narrative review are from previously reported studies and datasets, which have been cited within the manuscript.
